# Role of Peoxisome Proliferator Activator Receptor γ on Blood Retinal Barrier Breakdown

**DOI:** 10.1155/2008/679237

**Published:** 2008-01-27

**Authors:** Yasuo Yanagi

**Affiliations:** Department of Ophthalmology, School of Medicine, University of Tokyo, 7-3-1 Hongo, Bunkyo-ku, Tokyo 113-8655, Japan

## Abstract

The retinal vessels have two barriers: the retinal pigment epithelium and the retinal vascular endothelium. Each barrier exhibits increased permeability under various pathological conditions. This condition is referred to as blood retinal barrier (BRB) breakdown. Clinically, the most frequently encountered condition causing BRB breakdown is diabetic retinopathy. In recent studies, inflammation has been linked to BRB breakdown and vascular leakage in diabetic retinopathy. Biological support for the role of inflammation in early diabetes is the adhesion of leukocytes to the retinal vasculature (leukostasis) observed in diabetic retinopathy. PPARγ is a member of a ligand-activated nuclear receptor superfamily and plays a critical role in a variety of biological processes, including adipogenesis, glucose metabolism, angiogenesis, and inflammation. There is now strong experimental evidence to support the theory that PPARγ inhibits diabetes-induced retinal leukostasis and leakage, playing an important role in the pathogenesis of diabetic retinopathy. Therapeutic targeting of PPARγ may be beneficial to diabetic retinopathy.

## 1. BLOOD RETINAL BARRIER (BRB) BREAKDOWN IN DIABETIC RETINOPATHY

**Figure 1:**
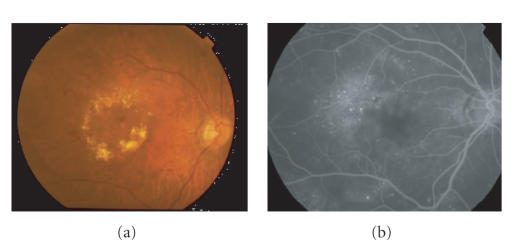
Macular edema in diabetic retinopathy. (a) Macular edema in diabetic retinopathy. (b) Increased vascular permeability is observed by fluorescin angiography. Note the leakage of the fluorescent dye showing the blood retinal barrier breakdown. Although the retinopathy is mild, this patient has a visual acuity of 20/200 due to severe macular edema.

The retinal vessels have a barrier consisting of the tight junction of the retinal pigment epithelium and the retinal vascular endothelium. Each barrier exhibits increased permeability under various pathological conditions. This condition is referred to as blood retinal barrier (BRB) breakdown. Clinically, the most frequently encountered condition that induces vascular permeability is diabetic retinopathy [1]. BRB breakdown causes retinal edema. Clinically, the retinal edema often affects macula, the highly sensitive area of the central retina, and often severely affects vision (Figure 1). The frequency of diabetic macular edema ranges from 2% to 13.3% of all diabetic patients, and 6.7% to 62% of insulin-dependent diabetic patients, and its incidence is 1.3% to 5.1% over a four-year observation period [2]. Due to the enhanced retinal vascular permeability, endothelial cell damage and capillary nonperfusion are aggravated. Much effort has been directed toward establishing effective treatments, and recent clinical studies have found that laser photocoagulation, pars plana vitrectomy, and antivascular endothelial growth factor (VEGF) therapy might be effective in ameliorating macular edema [3–6], but the treatment efficacy is limited and the results of the preliminary clinical investigation will have to be confirmed by further studies.

## 2. THE ROLE OF INFLAMMATION IN BRB BREAKDOWN

**Figure 2:**
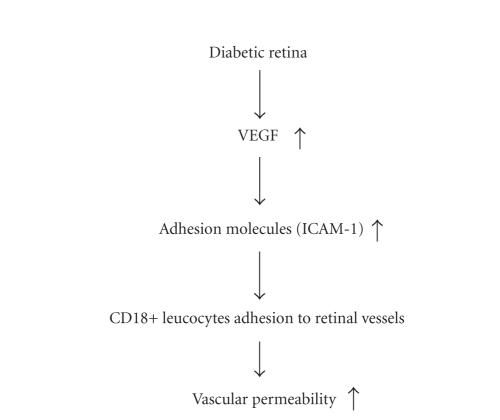
Schematic representation of the molecular mechanism of macular edema. VEGF drives the expression of ICAM-1 in the retinal vessels, which subsequently makes CD18+ leukocytes adherent to the retinal vessels. Adhesion of leukocytes to the retinal vessels leads to increased vascular leakage, subsequent endothelial cell damage, and capillary nonperfusion.

In recent studies, inflammation has been linked to vascular leakage in diabetic retinopathy [7]. Biological support for the role of inflammation in early diabetes is the adhesion of leukocytes to the retinal vasculature (leukostasis) observed in both experimental diabetic retinopathy in rats and in human diabetic retinopathy [8, 9]. Increased adhesion of leukocytes to the retinal vasculature is considered to promote vascular leakage. Thus, leukostasis is considered to be a critical event in the pathogenesis of diabetic retinopathy. Clinical investigations have demonstrated that the vitreous level of VEGF protein is higher in patients with diabetic macular edema than in patients with other conditions [10]. Ample evidence suggests that the adhesion of leukocytes to the retinal capillaries is controlled by vascular endothelial growth factor (VEGF), and focal adhesion molecules such as the intercellular adhesion molecule 1 (ICAM1) [11]. It is a commonly accepted molecular mechanism of leukocyte adhesion that VEGF drives the upregulation of the ICAM-1 molecule in the retinal endothelial cells [12, 13], and that this upregulated ICAM-1, together with upregulated leukocyte integrin CD18, triggers adhesion of leukocytes to the retinal vessels [14]. Indeed, CD18(−/−) and ICAM-1 (−/−) mice demonstrate significantly fewer adherent leukocytes in the retinal vasculature after the induction of diabetes with streptozotocin (STZ) [15]. It is, however, not only VEGF but also several other molecules that are involved in the expression of ICAM-1. NF-*κ*B molecules, activated by inflammation, also drive ICAM-1 expression [16]. Furthermore, blockage of the bioactivity of VEGF or ICAM-1 or inhibition of inflammatory pathways leads to decreased retinal leukocyte adhesion and reduced vascular leakage [17]. Thus, it is generally assumed that the upregulation of the adhesion molecule, triggered by VEGF and other inflammatory stimuli, is important in the leukostasis (Figure 2).

## 3. PPAR*γ* AND INFLAMMATION

**Figure 3:**
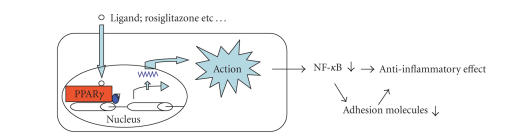
PPAR*γ* exerts anti-inflammatory effects. Schematic representation showing molecular pathways mediating the anti-inflammatory effects of PPAR*γ* ligands

PPAR*γ* is a member of a ligand-activated nuclear receptor superfamily and plays a critical role in a variety of biological processes, including adipogenesis, glucose metabolism, angiogenesis, and inflammation [18]. Synthetic ligands of PPAR*γ*, that is, thiazolidine derivatives such as rosiglitazone and pioglitazone, are used as oral antihyperglycemic agents for the therapy of non-insulin-dependent diabetes mellitus. In addition, recent studies have shown that PPAR*γ* ligands modulate the production of inflammatory mediators [19]. Actually, it has been reported that PPAR*γ* ligands, such as rosiglitazone and pioglitazone, suppress inflammatory diseases such as adjuvant-induced arthritis [19]. Importantly, some evidence suggests that PPAR*γ* is involved in the regulation of adhesion molecules. Previously, it has been demonstrated that PPAR*γ* ligand suppressed ICAM-1 expression in a murine model of intestinal ischemia-reperfusion injury [20] and in human umbilical vein endothelial cells in vitro [21]. Some of these anti-inflammatory functions are mediated through the inhibition of NF-*κ*B activation (Figure 3). Considering the close link between inflammation and diabetes, it is rational to consider that PPAR*γ* ligand therapy may also improve diabetic retinopathy.

## 4. PPAR*γ* IN BRB BREAKDOWN

**Figure 4:**
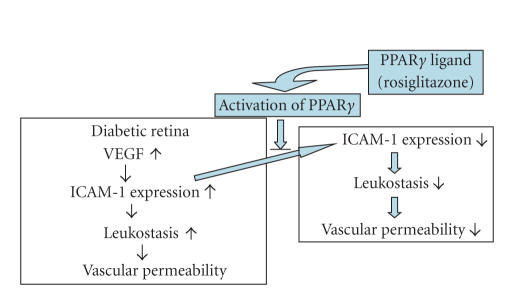
Involvement of PPAR*γ* ligand and its receptor system in retinal leukostasis and vacular permeability. Schematic representation showing the role of PPAR*γ* system in the retinal leukostasis and vascular permeability in diabetic retinopathy

We investigated the effects of a synthetic PPAR*γ* ligand, rosiglitazone, on an experimental diabetic model [22]. Additionally, heterozygous PPAR*γ*-deficient (+/-) mice were used in an experimental model to determine whether endogenous PPAR*γ* played a role [22]. Experimental diabetes was induced by intraperitoneal injection of STZ. This model is considered to destroy pancreatic beta cells completely [22] Retinal leukostasis quantification was performed by counting the number of adherent leukocytes after fluorescein-isothiocyanide (FITC)- Concanavalin A lectin (Con A) perfusion. A retinal leakage assay was performed by evaluating the retinal concentration of FITC-dextran after the animals were perfused. The results showed the PPAR*γ* agonist, rosiglitazone, inhibited both the retinal leukostasis and retinal leakage observed in the experimental diabetic rats and that the decreased expression of the endogenous PPAR*γ* in mice leads to the aggravation of retinal leukostasis and retinal leakage in diabetic mice. Together, these findings support the theory that the PPAR*γ* signaling pathway inhibits diabetes-induced retinal leukostasis and leakage. In addition, it was demonstrated that PPAR*γ* ligand suppresses ICAM-1 expression, but not VEGF expression, raising the possibility that NF-*κ*B mediated ICAM-1 is suppressed by PPAR*γ* ligand (Figure 4).

These results provide strong evidence to support the theory that PPAR*γ* activity plays an important role in the pathogenesis of diabetic retinopathy and introduce the novel possibility that the therapeutic targeting of PPAR*γ* may be beneficial to diabetic retinopathy.
